# Effects of Low-Frequency Electromagnetic Field on Oxidative Stress in Selected Structures of the Central Nervous System

**DOI:** 10.1155/2018/1427412

**Published:** 2018-12-13

**Authors:** Jan Budziosz, Agata Stanek, Aleksander Sieroń, Joanna Witkoś, Armand Cholewka, Karolina Sieroń

**Affiliations:** ^1^School of Health Sciences in Katowice, Department of Physical Medicine, Chair of Physiotherapy, Medical University of Silesia, Medyków Street 12, 40-752 Katowice, Poland; ^2^School of Medicine with the Division of Dentistry in Zabrze, Department of Internal Medicine, Angiology and Physical Medicine, Medical University of Silesia, Batorego Street 15, 41-902 Bytom, Poland; ^3^Department of Medical Physics, Chełkowski Institute of Physics, University of Silesia, 4 Uniwersytecka Street, 40-007 Katowice, Poland

## Abstract

**Objective:**

The aim of the study was to evaluate the effects of a 28-day exposure to a 50 Hz electromagnetic field of 10 kV*/*m on the oxidative stress in selected rat central nervous system (CNS) structures.

**Material and Methods:**

Twenty male Wistar rats served as experimental subjects. Ten rats were exposed to an electromagnetic field with a frequency of 50 Hz, intensity of 10 kV/m, and magnetic induction of 4.3 pT for 22 hours a day. The control group of ten rats was subject to sham exposure. Homogenates of the frontal cortex, hippocampus, brainstem, hypothalamus, striatum, and cerebellum were evaluated for selected parameters of oxidative stress.

**Results:**

Following the four-week exposure to a low-frequency electromagnetic field, the mean malondialdehyde levels and total oxidant status of CNS structures did not differ significantly between the experimental and control groups. However, the activities of antioxidant enzymes in brain structure homogenates were decreased except for frontal cortex catalase, glutathione peroxidase, and hippocampal glutathione reductase. The low-frequency electromagnetic field had no effect on the nonenzymatic antioxidant system of the examined brain structures except for the frontal cortex.

**Conclusion:**

The four-week exposure of male rats to a low-frequency electromagnetic field did not affect oxidative stress in the investigated brain structures.

## 1. Introduction

An electromagnetic field (EMF) occurs naturally in our environment. It is generated by geological structures in the Earth's crust, and all devices powered with alternating currents. Multihour use of such devices, including medical apparatus, results in a prolonged exposure to a low-frequency EMF (≤50 Hz). This may lead to disturbances in homeostasis and consequent disruption of the prooxidative-antioxidative balance within the central nervous system (CNS) of people permanently working in close proximity to devices generating electromagnetic fields [1].

In the last decade, the effects of a low-frequency electromagnetic field (LFEMF) on the human body have been referred to as electromagnetic sensitivity syndrome commonly associated with the rapid development of wireless technologies [2, 3]. The molecular mechanisms of nonthermal and nonionizing effects at low field intensities remain to be elucidated. Nevertheless, the number of research studies on this issue continues to grow [4–6]. A wide range of health problems associated with exposure to computer monitors, mobile phones, and other EMF-generating appliances has been reported. The most common symptoms include fatigue, irritation, headache, dryness of the skin and mucous membranes, sleep disturbance, and hormonal imbalance as well as cardiac and neural effects [7, 8]. Exposure to an EMF has also been linked with allergic reactions confirmed by a marked increase in the number of mast cells [9]. Numerous epidemiological studies have confirmed a significant increase in the risk of brain tumours [10, 11], parotid gland tumours [12], malignant melanoma [13], and lymphomas [14].

Several researchers have emphasised that exposure to the EMF might also cause increased reactive oxygen species (ROS) production and lead to oxidative stress [2, 15]. ROS have been implicated in the pathogenesis of neurodegenerative diseases including Alzheimer's disease, multiple sclerosis, or amyotrophic lateral sclerosis [16, 17]. It has also been found that oxidative stress might be involved in the development of Parkinson's disease [18, 19] and in the pathogenesis of mental disorders [20, 21]. Despite differences in the clinical features of neurodegenerative diseases, it has been suggested that they share common mechanisms such as dysregulation of iron metabolism, protein aggregation, oxidative stress, inflammatory processes, and mitochondrial dysfunction. However, all these phenomena have so far been studied separately without considering possible interactions or an option that they might occur as cascading events. Nevertheless, oxidative stress has been mentioned as the primary cause of neural cell damage [22].

The effect of the LFEMF on the prooxidative-antioxidative balance in CNS structures is yet to be extensively studied. Furthermore, only whole brain homogenates from experimental animals were evaluated. No assessment was carried out regarding particular brain structures, markers of oxidative stress, or activity of enzymatic and nonenzymatic antioxidant systems. Such incomplete analysis severely hinders the interpretation of results.

Therefore, the aim of this study was to evaluate the effects of a 28-day exposure to a 50 Hz electromagnetic field of 10 kV/m on the oxidative stress in selected CNS structures (frontal cortex, hippocampus, brainstem, hypothalamus, striatum, and cerebellum) of male rats.

## 2. Material and Methods

### 2.1. Animals

The study was performed with the approval no. 65/2008 of the Bioethical Committee for Animal Experimentation of the Medical University of Silesia in Katowice, Poland. All animals received humane care in compliance with the 8^th^ edition of the *Guide for the Care and Use of Laboratory Animals* published by the National Institute of Health [23].

Twenty male Wistar rats aged 10 weeks and weighing approximately 280 grams served as the experimental subjects. The animals were bred at the Institute of Experimental Medicine, Medical University of Silesia in Katowice Ligota, Poland. During the experiment, the rats were kept under optimal environmental conditions, i.e., at a temperature of 21°C and constant humidity, maintaining their 24-hour circadian rhythm. They were housed 10 per cage in plastic cages and fed with standard laboratory pellet food (Labofed B) and water at libitum.

### 2.2. Experimental Model

The rats were randomly divided into four groups, consisting of 10 animals each. Group A was exposed daily to an electromagnetic field with a frequency of 50 Hz, intensity of 10 kV/m, and magnetic induction of 4.3 pT. Exposure duration was 22 hours per day (with a break between 08 : 00 and 10 : 00). During the exposure, the animals remained in a plastic cage positioned between two electrodes placed 50 centimeters apart. The plastic cage did not impede the electromagnetic field and allowed the free movement of the rats. One electrode received a potential of 5 kV from a high-voltage transformer. The cage housing the rats was placed on the ground electrode. The control group of ten rats (group C) were subject to sham exposure (22 hours a day). Rats from group M were exposed for 28 successive days to an electromagnetic field with a frequency of 900 MHz generated by a mobile phone. Rats from group A + M were exposed, for 28 successive days, simultaneously to both a 50 Hz electromagnetic field and a radio-frequency electromagnetic field generated by a mobile phone. The physical parameters and duration of exposure were identical to those described for group A and group M.

In this manuscript, we have only presented the findings from group A and the control group.

Following the cycle of a 28-day exposure to an electromagnetic field and sham exposure, the rats were fasted for 24 hours and euthanized and the brains were removed. The dissection of brain structures was carried out by a person experienced in rat dissection. After decapitation and opening the skull, the brain was removed and the following structures were dissected: frontal cortex, striatum, hippocampus, hypothalamus, and cerebellum. For identifying brain structures, sections were systematically compared with images from the adult rat brain atlas [24]. The sampled tissues were frozen in solid carbon dioxide (dry ice), weighed, and kept deep-frozen at -70°C.

### 2.3. Preparation of Brain Tissue Homogenates

On the day of laboratory determinations, the tissues were homogenized for 3 minutes at 50 revolutions per minute using the Glas*-*Col Tissue Homogenizing System at a room temperature of 21°C. The tissues were homogenized in physiological buffered saline. The following were then determined in the obtained 5% tissue homogenates: total oxidant status (TOS), malondialdehyde (MDA), superoxide dismutase (SOD) and its isoenzymes (copper-zinc dismutase (SOD-CuZn), manganese dismutase (SOD-Mn)), catalase (CAT), glutathione peroxidase (GPx), glutathione reductase (GR), glutathione S-transferase (GST), and total antioxidant capacity (TAC).

### 2.4. Biochemical Analysis

#### 2.4.1. Oxidative Stress Marker Analysis

*(1) Determination of Prooxidative Status Parameters: Lipid Peroxidation Products and Total Oxidative Status*. The intensity of lipid peroxidation in the brain homogenates was measured spectrofluorimetrically as thiobarbituric acid-reactive substances (TBARS) according to Ohkawa et al. [25]. The TBARS concentrations were expressed as malondialdehyde (MDA) equivalents in *μ*mol/g protein. The inter- and intra-assay coefficients of variations (CV) were 2.2% and 8.4%, respectively.

The total oxidant status (TOS) was determined with the method described by Erel [26] and expressed in *μ*mol/g protein. The inter- and intra-assay coefficients of variations (CV) were 2.1% and 6.3%, respectively.

*(2) Determination of the Activity of Antioxidant Enzymes*. The superoxide dismutase (SOD-E.C.1.15.1.1) activity in brain homogenates was assayed using the Oyanagui method [27]. Enzymatic activity was expressed in nitrite unit (NU) in each mg of hemoglobin (Hb) or ml of blood plasma. One nitrite unit (1 NU) means a 50% inhibition of nitrite ion production by SOD in this method. SOD isoenzymes (SOD-Mn and SOD-CuZn) were measured using potassium cyanide as the inhibitor of the SOD-ZnCu isoenzyme. The inter- and intra-assay coefficients of variations (CV) were 2.8% and 5.4%, respectively.

Catalase (CAT-E.C.1.11.1.6.) activity was determined with the peroxide-purpald method developed by Johansson and Håkan Borg [28]. The method is based on the reaction of catalase with methanol in the presence of an optimal concentration of hydrogen peroxide. The obtained formaldehyde is measured spectrophotometrically at 550 nm with Purpald as a chromogen. The enzymatic activity of catalase was expressed in IU/g of protein. The inter- and intra-assay coefficients of variations (CV) were 2.6% and 6.1%, respectively.

The activity of brain homogenates' glutathione peroxidase (GPx-E.C.1.11.1.9.) was assayed using Paglia and Valentine's kinetic method [29], with t-butyl peroxide as a substrate and expressed as micromoles of NADPH oxidized per minute and normalised to one gram of protein [IU/g protein]. The inter- and intra-assay coefficients of variations (CV) were 3.3% and 7.4%, respectively.

The glutathione reductase activity (GR-E.C.1.6.4.2) in brain homogenates was assayed using Richterich's kinetic method [30], expressed as micromoles of NADPH utilized per minute, and normalised to one gram of protein [IU/g protein]. The inter- and intra-assay coefficients of variations (CV) were 2.2% and 5.7%, respectively.

The activity of glutathione S*-*transferase (GST-E.C.2.5.1.18) was determined with the kinetic method of Habig and Jakoby [31], expressed as micromoles of thioether formed per minute and normalised to one gram of protein [IU/g protein]. The inter- and intra-assay coefficients of variations (CV) were 3.4% and 7.3%, respectively.

*(3) Determination of Nonenzymatic Antioxidant Status.* Total antioxidant capacity (TAC) concentration in brain structure homogenates was determined with the method of Erel [32] based on oxidized ABTS (green in colour) decolorisation by antioxidants present in the sample and calibrated using Trolox and expressed in mmol/g protein. The inter- and intra-assay coefficients of variations (CV) were 1.1% and 3.8%, respectively.

### 2.5. Statistical Analysis

The obtained results were presented as the mean ± standard deviation (M ± SD) and analyzed using Statistica 7.1 PL software. All parameters of the group exposed to the electromagnetic field were compared to sham-exposed animals (control group). The Shapiro-Wilk test was used to test for normality of the distribution of particular variables. Intergroup differences were examined using a single-factor parametric ANOVA for quantitative variables. Additionally, the relationships identified by the ANOVA were verified using the NIR post hoc test for the particular groups. The level of statistical significance (*p* < 0.05) was used in all analyses.

## 3. Results

### 3.1. Oxidative Stress Parameters in the Frontal Cortex

In the frontal cortex homogenates from rats exposed to the LFEMF (group A), the mean activities of SOD, SOD-Mn, GPx, GST, and TOS were significantly lower, while the mean CAT activity was higher compared to the control rats (group C). However, the mean concentrations of MDA and TOS as well as the mean activities of SOD-CuZn and GR in rats exposed to the LFEMF did not differ significantly in comparison to the control group (Table 1).

### 3.2. Oxidative Stress Parameters in the Hippocampus

In the hippocampus homogenates from rats exposed to the low-frequency electromagnetic field, the mean activity of GR was significantly higher, while GST activity was significantly lower in comparison to the control group. However, no significant differences were observed between the mean MDA, TOS, and TAC concentrations as well as the mean SOD, SOD-Mn, SOD-CuZn, CAT, and GPx activities in rats exposed to the low-frequency electromagnetic field compared to the control group (Table 2).

### 3.3. Oxidative Stress Parameters in the Brainstem

In the brainstem homogenates from rats exposed to the LFEMF, the mean activities of SOD, SOD-Mn, SOD-CuZn, GR, and GST were significantly lower in comparison to those of the control rats. However, the mean MDA, TOS, and TAC concentrations as well as the mean CAT and GPx activities did not differ significantly in rats exposed to the LFEMF in comparison to the control group (Table 3).

### 3.4. Oxidative Stress Parameters in the Hypothalamus

In the hypothalamus homogenates from rats exposed to the LFEMF, the mean activity of GST homogenates was significantly higher compared to the control group. However, the mean MDA, TOS, and TAC concentrations as well as the mean SOD, SOD-Mn, SOD-CuZn, CAT, GPx, and GR activities in the hypothalamus homogenates from rats exposed to the LFEMF did not differ significantly in comparison to the sham-exposed rats (Table 4).

### 3.5. Oxidative Stress Parameters in the Striatum

In the striatum homogenates from rats exposed to the LFEMF, the mean activity of GST homogenates was significantly lower in comparison to the control group. The GST activity was the only parameter whose values differed significantly between the studied groups. However, the mean MDA, TOS, and TAC concentrations as well as the mean SOD, SOD-Mn, SOD-CuZn, CAT, GPx, and GR activities in the striatum homogenates from rats exposed to the LFEMF did not differ significantly in comparison to the sham-exposed rats (Table 5).

### 3.6. Oxidative Stress Parameters in the Cerebellum

In the cerebellum homogenates from rats exposed to the LFEMF, only the mean activity of GST homogenates was significantly lower in comparison to the control group. However, the mean MDA, TOS, and TAC concentrations as well as the mean SOD, SOD-Mn, SOD-CuZn, CAT, GPx, and GR activities in the group of rats exposed to the LFEMF did not differ significantly in comparison to the sham-exposed rats (Table 6).

## 4. Discussion

It has been found that the electromagnetic field may disrupt the prooxidative-antioxidative balance through increased ROS production, impaired ROS elimination, or the combined effect of both processes [33, 34]. Hence, the evaluation of oxidative stress severity must comprise the determination of oxidative stress markers as well as the activity of the enzymatic and nonenzymatic antioxidant systems simultaneously [35, 36].

Our study on the effects of a 4-week exposure of male rats to the LFEMF did not reveal any statistically significant differences in the mean MDA and TOS concentrations in CNS structures between the rats exposed to the LFEMF and sham-exposed animals. Hence, chronic exposure to the low-frequency electromagnetic field does not seem to have resulted in increased lipid peroxidation and ROS generation in the rat brain.

In the frontal cortex of rats exposed to the LFEMF, the mean activities of SOD and SOD-Mn homogenates were lower by 13.1% and 15.1%, respectively, compared to the control group. In addition, the mean activities of SOD, SOD-Mn, and SOD-CuZn in the brainstem of rats exposed to the LFEMF were lower by 16.5%, 15.9%, and 17.1%, respectively, in comparison to the control group. However, the mean SOD activity in the hippocampus, hypothalamus, striatum, and cerebellum in rats exposed to the LFEMF did not differ significantly in comparison to the control group. The mean CAT activity in the rats exposed to the LFEMF was only significantly higher by 77% in the frontal cortex compared to the control group. No significant differences in the mean CAT activity were found between the rats exposed to the LFEMF and the control group with respect to the examined remaining brain structures. In addition, the GPx activity in rats exposed to the LFEMF was lower by 23.6% than that of the control rats, but again, only in the frontal cortex. In the remaining examined brain structures, the GPx activity in rats exposed to the LFEMF did not differ significantly when compared to the control group. The mean GR activity in the rats exposed to the LFEMF was higher by 13.3% in the hippocampus, while in the brainstem its activity was lower by 25.1% in comparison to the control group. No significant differences in GR activity were found between the rats exposed to the LFEMF and the control group with respect to the remaining investigated brain structures. GST activity in rats exposed to the LFEMF was significantly lower in all investigated brain structures: in the frontal cortex by 32%, in the hippocampus by 14.2%, in the brainstem by 31.7%, in the hypothalamus by 23.2%, in the striatum by 11.5%, and in the cerebellum by 18.2% compared to the control group. Similarly, in our study, the mean TAC concentrations in the rats exposed to the LFEMF were lower by 13.4% in comparison to the control group, but only in the frontal cortex. No significant differences in TAC concentrations were found between the rats exposed to the LFEMF and the control group with respect to the remaining investigated brain structures.

Different responses of oxidative stress parameters in brain structures under investigation might have been caused by the different impact of the LFEMF on these structures as well as differences in their functions.

There are only a few reports available on the effects of the LFEMF on prooxidative-antioxidative balance within the central nervous system, but it should be noted that, due to a high level of aerobic metabolism, large amounts of unsaturated fatty acids, and lower antioxidant activity, neurons are particularly vulnerable to disturbances in the prooxidative-antioxidative balance [37, 38].

The available results of researches are not unequivocal, which might be due to the differences in the physical parameters of applied electromagnetic fields and different methodologies of exposure. Jelenković et al. [39] are the only investigators who have evaluated the effects of exposure to the LFEMF on several brain structures in rats. However, the nonenzymatic antioxidant system was not assessed, and hence, no ultimate conclusions can be drawn regarding the prooxidative-antioxidative balance. Also, the rats were exposed to the LFEMF (50 Hz, 0.5 mT) for 7 days only. Nevertheless, the production of superoxide anion radical and MDA concentrations increased in all CNS-investigated structures. A significant increase in nitric oxide production was found in the frontal cortex and hypothalamus, while higher SOD activity was only observed in the hypothalamus.

Akdag et al. [40] examined brain homogenates of rats exposed to a 100 or 500 *μ*T electromagnetic field for 2 hours a day for 10 months. The CAT activity decreased in both exposure groups. The TAC concentration was lower in the 500 *μ*T group compared to the 100 *μ*T and sham-exposed groups, while MDA, TOS concentration, and oxidative stress index were higher.

Lee et al. [41] observed a significant increase in chemiluminescence and SOD activity in brain homogenates of mice after a 3-hour exposure to a 60 Hz electromagnetic field.

Falone et al. [42] showed that continuous 10-day exposure to a 50 Hz, 0.1 mT EMF significantly affected the antioxidative capacity of the female rat brain, the effect being age-dependent. The activity of antioxidant enzymes increased in young and decreased in old animals. This observation was supported by the results of Rageh et al. [43], who exposed 10-day-old rats to a continuous 50 Hz, 0.5 mT electromagnetic field for 30 days. They found that EMF exposure resulted in higher MDA concentration and SOD activity and increased the rate of oxidative damage to cellular DNA. It should be emphasized though that the above study was performed using juvenile rats whose CNS had not been fully developed yet.

Physical factors of limited intensity might also have some beneficial effects in the form of adaptive process stimulation. Ciejka et al. [44] observed that a longer exposure to a 7 mT, 40 Hz electromagnetic field apparently resulted in the adaptation to experimental conditions. An analysis of brain homogenates of adult rats after a 60-minute daily exposure to the electromagnetic field over a period of 10 days revealed a significant increase in the sulfhydryl group and protein concentration, while a 30-minute daily exposure caused a significant increase in lipid peroxidation.

The exposure of our experimental rats to a 50 Hz EMF lasted for four weeks. Since human exposure to the EMF is typically long-term, we did not aim to examine the effects of short-term exposure. The issue is challenging and requires further studies.

## 5. Conclusions

Summing up, it can be concluded that a four-week exposure of male rats to the low-frequency electromagnetic field does not affect oxidative stress in the studied brain structures.

## Figures and Tables

**Table 1:** Concentrations of oxidative stress biomarkers: malondialdehyde (MDA) and total oxidant status (TOS); activity of antioxidant enzymes: superoxide dismutase (SOD) and isoenzymes (SOD-Mn, SOD-CuZn), catalase (CAT), glutathione peroxidase (GPx), glutathione reductase (GR), and glutathione S*-*transferase (GST); and concentration of nonenzymatic antioxidants—total antioxidant capacity (TAC) in the frontal cortex homogenates from rats exposed to low-frequency electromagnetic field (group A) and sham-exposed rats (control group) (group C) with results of ANOVA for all examined groups (A, C, M, and A + M) and post hoc NIR test for two selected groups: A and C.

	Group A	Group C	ANOVA results	
Parameter	M ± SD	M ± SD	*F*; *p* value	*p* value of NIR test
MDA concentration (*μ*mol/g protein)	1.12 ± 0.09	1.04 ± 0.16	9.68; **<0.001**	0.538
TOS concentration (*μ*mol/g protein)	2.17 ± 0.35	2.10 ± 0.39	0.6; 0.622	0.623
SOD activity (NU/mg protein)	49.38 ± 5.85	56.80 ± 3.25	13.02; **<0.001**	**<0.001**
SOD-Mn activity (NU/mg protein)	29.75 ± 3.12	35.05 ± 2.53	13.02; **<0.001**	**<0.001**
SOD-CuZn activity (NU/mg protein)	19.63 ± 4.45	21.75 ± 2.19	2.82; 0.040	0.219
CAT activity (IU/g protein)	6.16 ± 0.92	3.48 ± 1.05	17.66; **<0.001**	**<0.001**
GPx activity (IU/g protein)	0.59 ± 0.09	0.77 ± 0.13	3.87; 0.017	**<0.01**
GR activity (IU/g protein)	18.96 ± 1.98	19.91 ± 1.28	14.81; **<0.001**	0.097
GST activity (IU/g protein)	2.03 ± 0.21	2.99 ± 0.21	47.03; **<0.001**	**<0.01**
TAC concentration (mmol/g protein)	0.06 ± 0.00	0.07 ± 0.01	3.34; **0.031**	**<0.01**

**Table 2:** Concentrations of oxidative stress biomarkers: malondialdehyde (MDA) and total oxidant status (TOS); activity of antioxidant enzymes: superoxide dismutase (SOD) and isoenzymes (SOD-Mn, SOD-CuZn), catalase (CAT), glutathione peroxidase (GPx), glutathione reductase (GR), and glutathione S*-*transferase (GST); and concentration of nonenzymatic antioxidants—total antioxidant capacity (TAC) in the hippocampus homogenates from rats exposed to low-frequency electromagnetic field (group A) and sham-exposed rats (control group) (group C) with results of ANOVA for all examined groups (A, C, M, and A + M) and post hoc NIR test for two selected groups: A and C.

	Group A	Group C	ANOVA results	
Parameter	M ± SD	M ± SD	*F*; *p* value	*p* value of NIR test
MDA concentration (*μ*mol/g protein)	1.79 ± 0.48	1.56 ± 0.34	1.86; 0.156	0.294
TOS concentration (*μ*mol/g protein)	2.68 ± 0.43	2.36 ± 0.23	1.50; 0.232	0.081
SOD activity (NU/mg protein)	70.73 ± 9.09	65.78 ± 6.63	8.78; **0.035**	1.000
SOD-Mn activity (NU/mg protein)	38.75 ± 3.87	37.05 ± 3.02	13.66; **<0.001**	0.278
SOD-CuZn activity (NU/mg protein)	31.98 ± 6.64	28.73 ± 6.37	1.06; 0.379	0.212
CAT activity (IU/g protein)	5.02 ± 0.78	5.79 ± 0.76	14.40; **<0.001**	0.084
GPx activity (IU/g protein)	0.45 ± 0.19	0.47 ± 0.13	0.20; 0.898	0.848
GR activity (IU/g protein)	30.56 ± 3.34	26.97 ± 2.19	11.56; **<0.001**	**<0.01**
GST activity (IU/g protein)	3.92 ± 0.37	4.57 ± 0.28	6.96; **<0.001**	**<0.01**
TAC concentration (mmol/g protein)	0.10 ± 0.01	0.09 ± 0.01	0.38; 0.765	0.612

**Table 3:** Concentrations of oxidative stress biomarkers: malondialdehyde (MDA) and total oxidant status (TOS); activity of antioxidant enzymes: superoxide dismutase (SOD) and isoenzymes (SOD-Mn, SOD-CuZn), catalase (CAT), glutathione peroxidase (GPx), glutathione reductase (GR), and glutathione S*-*transferase (GST); and concentration of nonenzymatic antioxidants—total antioxidant capacity (TAC) in the brainstem homogenates from rats exposed to low-frequency electromagnetic field (group A) and sham-exposed rats (control group) (group C) with results of ANOVA for all examined groups (A, C, M, and A + M) and post hoc NIR test for two selected groups: A and C.

	Group A	Group C	ANOVA results	
Parameter	M ± SD	M ± SD	*F*; *p* value	*p* value of NIR test
MDA concentration (*μ*mol/g protein)	1.93 ± 0.46	2.60 ± 0.38	3.12; **0.020**	0.568
TOS concentration (*μ*mol/g protein)	4.04 ± 1.25	3.19 ± 1.36	1.14; 0.349	0.217
SOD activity (NU/mg protein)	120.63 ± 5.58	144.45 ± 9.87	16.81; **<0.001**	**<0.001**
SOD-Mn activity (NU/mg protein)	63.79 ± 4.43	75.84 ± 6.37	14.76; **<0.001**	**<0.01**
SOD-CuZn activity (NU/mg protein)	56.84 ± 4.05	68.61 ± 8.48	17.26; **<0.001**	**<0.01**
CAT activity (IU/g protein)	11.64 ± 1.40	12.81 ± 1.10	14.26; **<0.01**	0.932
GPx activity (IU/g protein)	1.14 ± 0.31	1.38 ± 0.17	1.72; 0.181	0.103
GR activity (IU/g protein)	21.68 ± 1.55	28.95 ± 1.24	38.32; **<0.001**	**<0.001**
GST activity (IU/g protein)	3.77 ± 0.25	5.52 ± 0.54	34.71; **<0.001**	**<0.001**
TAC concentration (mmol/g protein)	0.12 ± 0.02	0.11 ± 0.04	0.57; 0.636	0.304

**Table 4:** Concentrations of oxidative stress biomarkers: malondialdehyde (MDA) and total oxidant status (TOS); activity of antioxidant enzymes: superoxide dismutase (SOD) and isoenzymes (SOD-Mn, SOD-CuZn), catalase (CAT), glutathione peroxidase (GPx), glutathione reductase (GR), and glutathione S*-*transferase (GST); and concentration of nonenzymatic antioxidants—total antioxidant capacity (TAC) in the hypothalamus homogenates from rats exposed to low-frequency electromagnetic field (group A) and sham-exposed rats (control group) (group C) with results of ANOVA for all examined groups (A, C, M, and A + M) and post hoc NIR test for two selected groups: A and C.

	Group A	Group C	ANOVA results	
Parameter	M ± SD	M ± SD	*F*; *p* value	*p* value of NIR test
MDA concentration (*μ*mol/g protein)	1.03 ± 0.15	0.76 ± 0.13	11.76; <**0.001**	0.079
TOS concentration (*μ*mol/g protein)	2.85 ± 0.51	2.68 ± 0.35	1.37; 0.268	0.535
SOD activity (NU/mg protein)	72.00 ± 12.56	82.99 ± 7.58	1.40; 0.260	0.064
SOD-Mn activity (NU/mg protein)	48.53 ± 5.67	52.41 ± 4.11	0.73; 0.543	0.263
SOD-CuZn activity (NU/mg protein)	23.46 ± 9.42	30.59 ± 3.76	6.48; **0.029**	0.119
CAT activity (IU/g protein)	9.11 ± 2.78	8.80 ± 1.73	0.28; 0.841	0.800
GPx activity (IU/g protein)	0.47 ± 0.44	0.43 ± 0.30	0.54; 0.660	0.862
GR activity (IU/g protein)	23.91 ± 3.14	24.05 ± 2.22	2.69; **0.046**	0.921
GST activity (IU/g protein)	4.86 ± 0.94	6.33 ± 0.49	11.18; **<0.001**	**<0.001**
TAC concentration (mmol/g protein)	0.09 ± 0.01	0.10 ± 0.01	1.03; 0.393	0.652

**Table 5:** Concentrations of oxidative stress biomarkers: malondialdehyde (MDA) and total oxidant status (TOS); activity of antioxidant enzymes: superoxide dismutase (SOD) and isoenzymes (SOD-Mn, SOD-CuZn), catalase (CAT), glutathione peroxidase (GPx), glutathione reductase (GR), and glutathione S*-*transferase (GST); and concentration of nonenzymatic antioxidants—total antioxidant capacity (TAC) in the striatum homogenates from rats exposed to low-frequency electromagnetic field (group A) and sham-exposed rats (control group) (group C) with results of ANOVA for all examined groups (A, C, M, and A + M) and post hoc NIR test for two selected groups: A and C.

	Group A	Group C	ANOVA results	
Parameter	M ± SD	M ± SD	*F*; *p* value	*p* value of NIR test
MDA concentration (*μ*mol/g protein)	1.02 ± 0.12	1.12 ± 0.26	1.86; 0.156	0.278
TOS concentration (*μ*mol/g protein)	1.64 ± 0.99	1.75 ± 0.86	14.70; **<0.01**	1.000
SOD activity (NU/mg protein)	64.35 ± 15.38	64.16 ± 8.73	16.16; 0.201	1.000
SOD-Mn activity (NU/mg protein)	32.49 ± 3.87	32.78 ± 2.31	4.60; **<0.01**	0.487
SOD-CuZn activity (NU/mg protein)	31.86 ± 13.15	31.39 ± 8.29	1.13; 0.350	0.908
CAT activity (IU/g protein)	4.73 ± 2.12	3.79 ± 1.20	55.86;**<0.001**	0.277
GPx activity (IU/g protein)	1.15 ± 0.22	1.25 ± 0.15	6.16; **<0.01**	0.238
GR activity (IU/g protein)	25.92 ± 3.56	25.55 ± 2.76	21.42; **<0.001**	1.000
GST activity (IU/g protein)	2.93 ± 0.34	3.32 ± 0.19	6.63; **<0.01**	**<0.01**
TAC concentration (mmol/g protein)	0.08 ± 0.01	0.08 ± 0.01	0.36; 0.783	0.347

**Table 6:** Concentrations of oxidative stress biomarkers: malondialdehyde (MDA) and total oxidant status (TOS); activity of antioxidant enzymes: superoxide dismutase (SOD) and isoenzymes (SOD-Mn, SOD-CuZn), catalase (CAT), glutathione peroxidase (GPx), glutathione reductase (GR), and glutathione S*-*transferase (GST); and concentration of nonenzymatic antioxidants—total antioxidant capacity (TAC) in the cerebellum homogenates from rats exposed to low-frequency electromagnetic field (group A) and sham-exposed rats (control group) (group C) with results of ANOVA for all examined groups (A, C, M, and A + M) and post hoc NIR test for two selected groups: A and C.

	Group A	Group C	ANOVA results	
Parameter	M ± SD	M ± SD	*F*; *p* value	*p* value of NIR test
MDA concentration (*μ*mol/g protein)	3.30 ± 1.269	2.76 ± 0.79	13.73; **<0.001**	0.292
TOS concentration (*μ*mol/g protein)	4.14 ± 0.58	4.40 ± 0.76	14.82; **<0.01**	1.000
SOD activity (NU/mg protein)	99.80 ± 16.56	94.77 ± 4.84	21.23; **<0.01**	1.000
SOD-Mn activity (NU/mg protein)	97.71 ± 14.55	92.05 ± 4.12	20.89; **<0.001**	1.000
SOD-CuZn activity (NU/mg protein)	2.28 ± 2.17	2.71 ± 1.21	17.86; **<0.001**	1.000
CAT activity (IU/g protein)	12.38 ± 3.42	9.74 ± 2.96	4.07; **0.014**	0.129
GPx activity (IU/g protein)	1.88 ± 0.23	1.79 ± 0.23	13.01; **<0.01**	1.000
GR activity (IU/g protein)	19.08 ± 3.36	18.93 ± 1.11	15.62; **<0.01**	1.000
GST activity (IU/g protein)	2.93 ± 0.42	3.58 ± 0.53	18.39; **<0.001**	**<0.01**
TAC concentration (mmol/g protein)	0.07 ± 0.06	0.13 ± 0.01	20.32; **<0.001**	1.000

## Data Availability

All data are included in the tables in the article.
